# Determination of arsenic and lead in single hair strands by laser ablation inductively coupled plasma mass spectrometry

**DOI:** 10.1038/s41598-017-03660-6

**Published:** 2017-06-13

**Authors:** Ruxin Luo, Xiaohong Su, Weicong Xu, Sujing Zhang, Xianyi Zhuo, Dong Ma

**Affiliations:** 1 Shanghai Key Laboratory of Forensic Medicine, Shanghai Forensic Service Platform, Institute of Forensic Science, Ministry of Justice, P.R.China, Shanghai, 200063 Shanghai China; 2 Lanzhou Industry Research Institude, Lanzhou, 730000 China; 3 Department of Health Inspection and Quarantine, School of Public Health, Southern Medical University, Key Laboratory of Military Preventive Medicine of Guangdong Province, Guangzhou, 510515 China

## Abstract

The purpose of this study was to develop matrix-matched hair standards and a LA-ICP-MS technique for determination of the As and Pb in a single human hair using single spot scan mode. These results could subsequently be used to infer when the element entered the body. This study was conducted in two parts. First, a method was developed and validated for the elemental analysis of hair by LA-ICP-MS. A calibration strategy in LA-ICP-MS was developed using prepared matrix-matched laboratory hair standards doped with analytes of interest at a defined concentration. The use of hair strand standards enables calibration curves to be obtained by plotting the analyte ion (M^+^) intensity normalized to^34^S^+^(the ratio M^+^/^34^S^+^) as a function of the concentration determined by ICP-MS of the acidic digests. The linear correlation coefficients (R^2^) of the calibration curves for the analytes As and Pb were typically between 0.9970 and 0.9998, respectively. Second, an actual hair was measured using the developed method. The spatial distribution of As along the hair was observed in a hair sample from a leukaemia patient treated with arsenic trioxide (As_2_O_3_). The actual and estimated times over which the drug entered the body were compared and discussed.

## Introduction

Hair analysis for essential and toxic metals is of increasing importance in studies related to medicine^1, 2^, forensics^3^, archaeology^4, 5^and nutrition^6^. The analysis of hair has certain advantages, including the simplicity of the matrix and the ease of sampling, transfer, and storage^7^. Another advantage of hair analysis is the fact that the obtained results are not the result of any homeostatic mechanisms, unlike assays carried out on blood samples^8^. Moreover, human hair grows at a rate of approximately 1 cm per month, and the level of the different elements found in hair is reflective of their levels in the body medium from which the hair was formed. Thus, hair samples provide a historical record of elements assimilated from the environment^9^.

Elements at trace concentration levels have been quite often determined in bulk hair samples after acid digestion using inductively coupled plasma mass spectrometry (ICP-MS)^10^. However, this analytical approach may not provide sufficient information because it ignores the spatial distribution of metals in the analysed hair strands. To date, researching the spatial distribution of metals in biological samples or in tissue sections is most commonly performed using three sensitive mass spectrometric techniques: secondary ion mass spectrometry (SIMS)^11^, particle induced x-ray emission (PIXE)^12^ and laser ablation inductively coupled plasma mass spectrometry (LA-ICP-MS)^13^.

LA-ICP-MS has been established as a suitable technique for quantitative imaging of metals in biological tissues because of its high-sensitivity, temporal elemental analysis capability, minimal sample destruction, high resolution, and direct solid-state sampling^4^. As with other solid sampling analysis techniques, laser ablation provides challenges with respect to calibration. Nevertheless, several quantification strategies have been developed for analysis of the elemental distribution of human hair using LA-ICP-MS, such as the use of certified reference materials (CRM) or the preparation of matrix-matched laboratory standards^1^.

The most frequently used ablation mode is single line scan. However, the superposition of pulse signal may deteriorate the spatial resolution of the elements in human hair during the line scan. Single spot scan has a higher spatial resolution, because there is enough intersite pause between two spots. So single spot scan mode can more clearly show the distribution of elements in the hair.

The aim of this work was to develop matrix matched hair standards and a LA-ICP-MS technique for determination of the As and Pb in a single human hair using single spot scan mode. At the same time, the As along the hair of a person who had been treated with arsenic trioxide (As_2_O_3_) in leukaemia was monitored in a single hair strand using LA-ICP-MS. These results established a good foundation for studying when drugs enter the body.

## Experimental

### Instrumentation

An Agilent 7500 Ce inductively coupled plasma mass spectrometer (Agilent Technologies, Tokyo, Japan) operating in standard mode was coupled with a UP 213 New Wave laser ablation system-wavelength of the Nd:YAG laser:213 nm (Cambridge, UK). LA-ICP-MS uses a laser beam to ablate sample material in a helium atmosphere under normal pressure in the laser ablation chamber. The ablated sample was transported into ICP-MS by a helium gas stream. A standard reference material (NIST 612) was used only to tune the ablation system. For optimization, laser parameters were varied to obtain the highest analyte ion intensities. The experimental parameters (RF power: 1500 W and carrier gas flow rate: 0.87 L/min) of the LA-ICP-MS measurements were optimized to obtain the maximum analyte ion (M^+^) intensity and minimum intensity of the oxide (MO^+^) and double charged (M^2+^) ions. Details of the instrumentation and experimental parameters are shown in Table 1.

**Table 1:** Instrumental parameters using an ICP-MS and laser ablation system.

RF power (W)	1500
Carrier gas (L min^−1^)	0.87
Isotopes monitored	^34^S, ^75^As, ^208^Pb
Laser ablation mode	Single spot scan
Dwell time (s)	6
Intersite pause (s)	20
Repetition frequency (Hz)	10
Spot size	55 um
Laser power density (J/cm^2^)	6.89

### Reagents

Ultrapure 65% HNO_3_ (Merck, Darmstadt, Germany) and 29–32% hydrogen peroxide (Alfa Aesar, USA) were used in this study. High-purity deionized water (resistivity 18.2 MΩ cm) obtained using a Milli-Q laboratory water-purification system (Millipore, Massachusetts, USA) was used throughout. All solutions were stored in high-density polyethylene bottles. Single-element standard solutions (Pb and As) at concentrations of 1000 mg/L were purchased from NSI Solutions (Raleigh, NC, USA).

### Samples and sample preparation

Hair samples provided by a patient (female, 48 kg) who had been treated with arsenic trioxide (As_2_O_3_) in acute promyelocytic leukaemia were analysed to verify the applicability of the proposed method and infer when the drug entered the body.

The patient’s hair was labelled with hair dye before beginning medication. After therapy, the patient’s hair near dyed hair was cut close to the root in the scalp, washed with acetone and deionised water, and dried at room temperature. Single hair strands were fixed one by one onto double sided adhesive tape and analysed by LA-ICP-MS.

### Ethics statement

The subject agreed to participate in this study and was informed that arsenic would be determined in her hair sample. This study was approved by the Ethics Committee of the Institute of Forensic Science, Ministry of Justice, PR China, and written informed consent was obtained from all participants. All experimental methods involving human participants were in accordance with the 1975 Declaration of Helsinki.

### Preparation of matrix-matched laboratory hair standards

To prepare the matrix-matched standards, tufts of hair were taken from a volunteer without any history of As and Pb exposure. Hair was washed according to the method proposed by Puchyr, with a 1:200 v/v dilution of Triton X-100, deionized water and acetone^14^. After washing, the samples were dried in an oven at 75 ± 5 °C. Subsequently, sub-samples of the hair were incubated in different As and Pb solutions containing 5–100 mg L^−1^ of As and 0.05–5 mg L^−1^ of Pb for 24 h at room temperature. After this period, the hair was removed, thoroughly washed with Milli-Q water, left to dry, and then cut into about approximately 2 mm strands. Quality control hair strands at two different concentration were made according to the procedure described above.

A subset of the strands was used in the direct laser ablation, while the other part was acid-digested^10^. Samples (20 mg) were placed in 15-mL polypropylene tubes (Corning, New York, USA), and 0.8 mL HNO_3_ (Merck, Darmstadt, Germany) and 0.2 mL H_2_O_2_ (Alfa Aesar, USA) were subsequently added. Then, the samples were digested at 90 °C for 3 h and diluted to 10 ml with high-purity deionized water.

### Calibration strategy and sample analysis

Corrections for variations in ablation efficiency and plasma variation were made by internal standardization using the matrix element sulphur (^34^S). A calibration curve was obtained by plotting the observed ratio of analyte ion intensities to ^34^S^+^ intensities (obtained by direct ablation of the strands) versus the accurate metal concentration determined by ICP-MS. Each point of the calibration curve was the average signal obtained by ablating at least twenty hair strands (one laser spot for a hair strand). The RSD of each point was less than 15%. The limit of detection (LOD) was calculated by the ablation of 10 washed native unexposed hair strands^15^.

**Figure 1:**
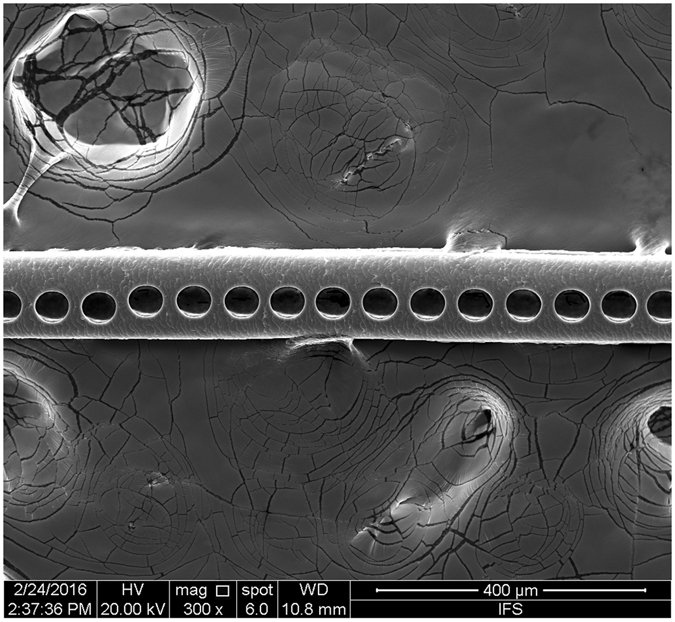
Post-ablation photo of hair sample.

The hair sample was ablated by single spot scan mode. The diameter of the spot was 55 μm, and the distance between two spots was 15 μm. The ablated hair was photographed using FEI Quanta 650 scanning electron microscope (FEI, Czech Republic) (Fig. 1).

## Results and Discussion

### Development of a LA-ICP-MS method for analysis of arsenic and lead in hair

**Figure 2:**
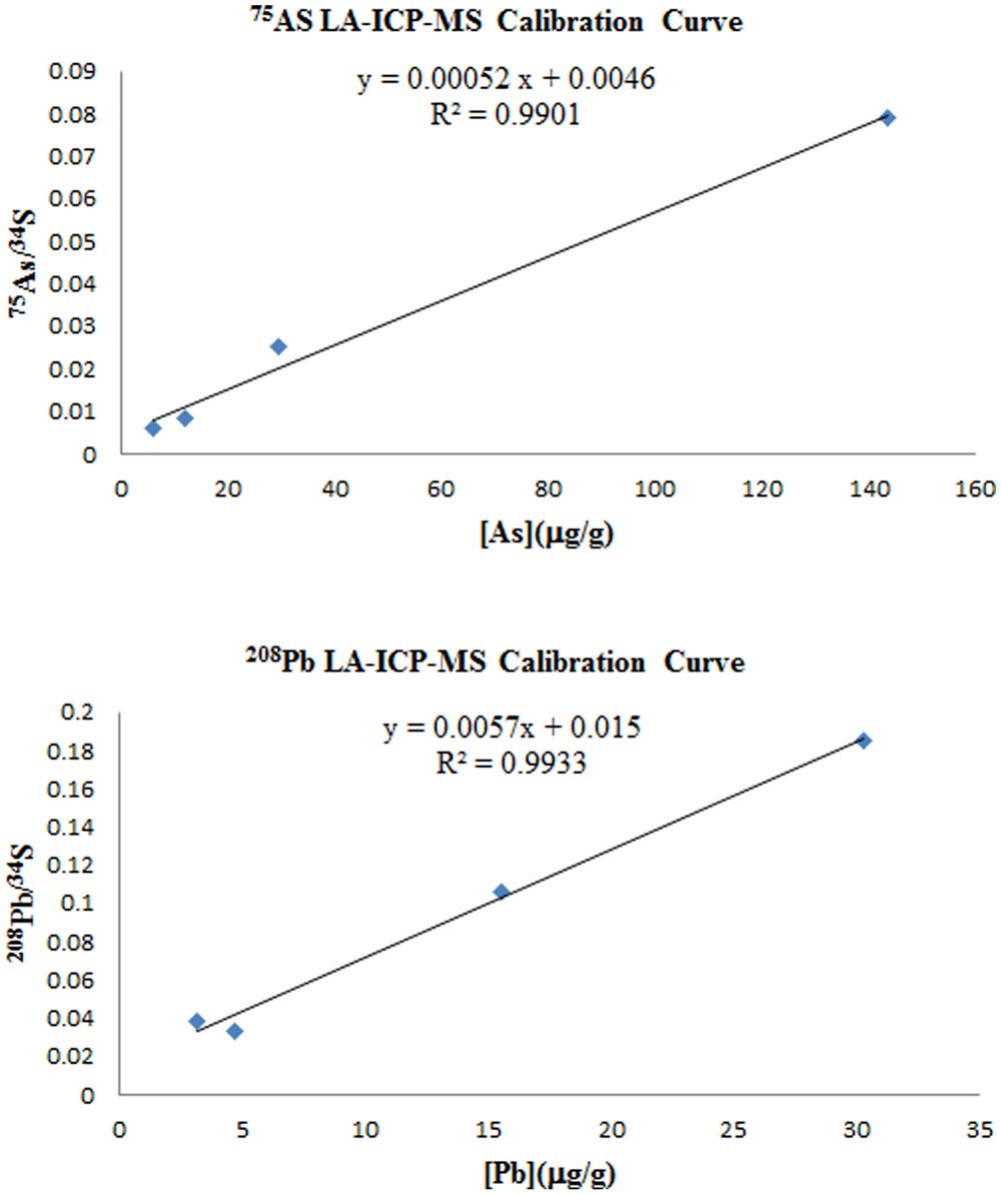
Arsenic and lead calibration curve from the analysis of ten in-house hair standards (R2 = 0.9901 and 0.9933).

**Figure 3:**
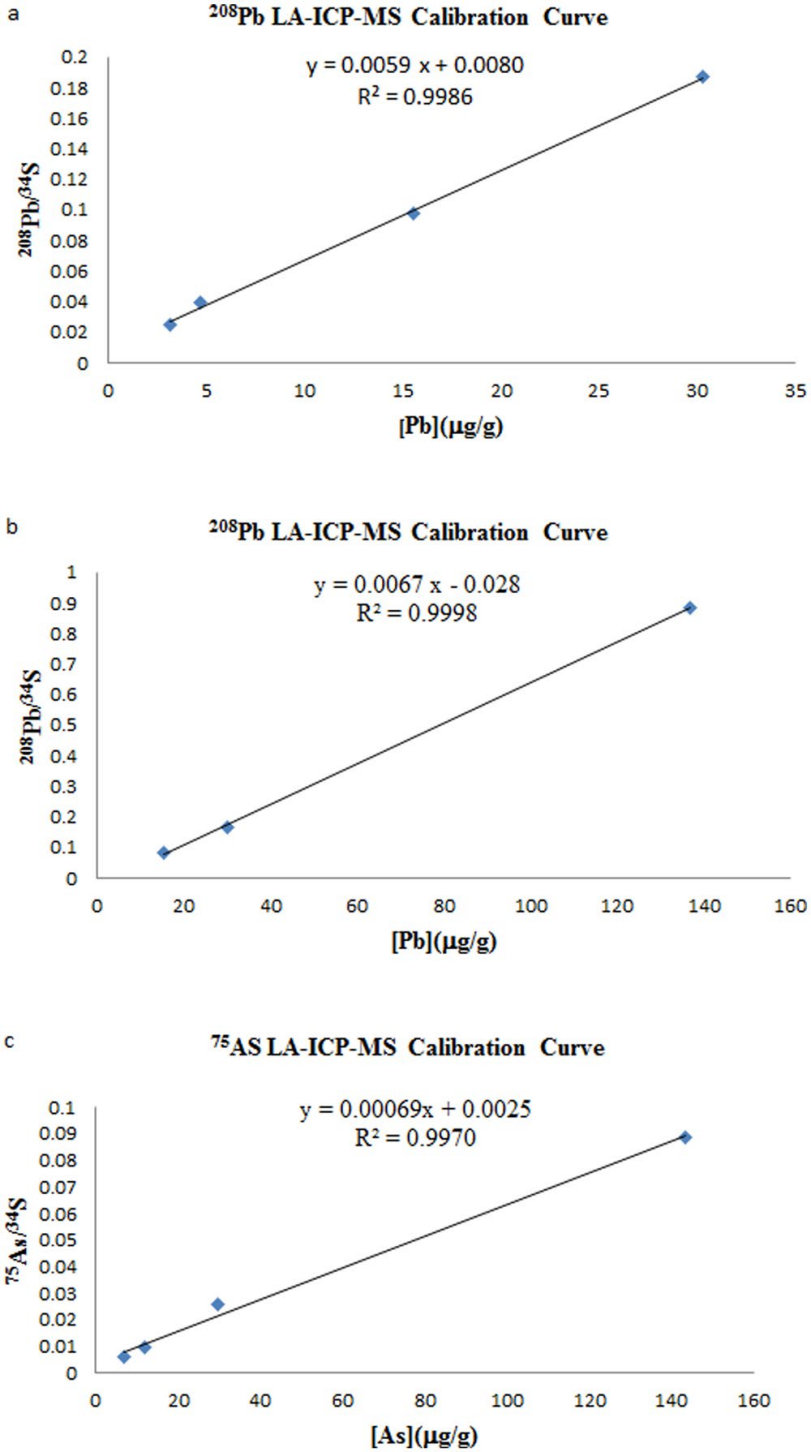
(**a**) Calibration curve and respective parameters obtained using Pb-enriched hair strands, concentration range: 3.21–30.3 mg/g. (**b**) Calibration curve and respective parameters obtained using Pb-fenriched hair strands, concentration range: 15.54–136.79 mg/g. (**c**) Calibration curve and respective parameters obtained using As-enriched hair strands, concentration range: 6.91–134.5 mg/g.

To quantify the elemental distributions of hair samples using LA-ICP-MS data, a set of metal-enriched hair strands was prepared. The quantification of As and Pb in the metal-enriched hair strands was performed by ICP-MS using liquid nebulization. The laser ablation system was optimized using the NIST 612 Trace Elements in Glass standard reference material, after which ten metal-enriched hair standards were analysed by LA-ICP-MS. The previously determined element concentrations in the hair were plotted against the ^75^As^+^(^208^Pb^+^)/^34^S^+^ signal intensity ratios, and the linear calibration curve had a correlation coefficient of 0.9901 for As (6.91–134.5 μg/g) and 0.9933 for Pb (3.21~30.3 μg/g) (Fig. 2). The same calibration procedure was later used with twenty metal-enriched hair standards analysed by LA-ICP-MS. The previously determined concentrations were plotted against the ^75^As^+^(^208^Pb^+^)/^34^S^+^ signal intensity ratios, and the calibration curve had a correlation coefficient of 0.9970 for As(6.91–134.5 μg/g) and 0.9986 and 0.9998 for Pb (3.21–30.3 μg/g and 15.54–136.79 μg/g, respectively) (Fig. 3). These results indicated that smaller sample size resulted in poorer correlation coefficients. This result was due to differences in the amount of each element adsorbed onto the hair during the incubating process. Thus, the sample size was increased as much as possible to better represent the population sample. The calibration curves in our study obtained showed good linearity with correlation coefficients between 0.9970 and 0.9998 for As and Pb as summarized in Fig. 3. Their linearity was similar to others reported for LA-ICP-MS, or even better. Usarat Kumtabtima *et al*.^15^ carried out LA-ICP-MS measurements of hair strands using two different types of standards. The linear correlation coefficient of As and Pb therein were 0.9910 and 0.9967, respectively. The limits of detection of As and Pb in a single hair strand were 2.1 and 1.3 μg/g, respectively. The LOQ’s were the lowest concentrations used for the calibration of each element.

To check the precision and accuracy of the developed LA-ICP-MS method for element quantification in hair, quality control hair strands were analysed by LA-ICP-MS. To assess the accuracy of this method for determination of As and Pb in a single human hair by LA-ICP-MS, quality control hair strands were digested in a microwave oven. The analytes were then measured by ICP-MS, and this value was treated as the reference value. The precision of the measurements was ascertained by performing three runs for the quality control hair strands on three days. Accuracy and precision figures were given in Table 2. Good agreement was found between the results obtained by LA-ICP-MS and ICP-MS for the same elements as shown in Table 2. According to Table 2, the standard deviation observed for LA-ICP-MS was in general larger than that observed for ICP-MS. This increase occurred because the element concentration varied along the hair, which was not detected in the analysis using ICP-MS^16^.

**Table 2:** Comparison of analyte concentration in quality control hair strands measured by LA-ICP-MS (via the calibration curve obtained from matrix matched hair standards) with the values measured by ICP-MS. Values in μg/g.

Element	Quality control hair strands	ICP-MS	LA-ICP-MS	Accuracy(%)	Inter-day (n = 3)
As	QC1	29.02 ± 3.35	26.32 ± 4.46	90.70%	2.53%
	QC2	134.5 ± 2.23	144.5 ± 8.58	107.43%	5.56%
Pb	QC1	9.18 ± 0.30	8.99 ± 1.94	97.91%	1.49%
	QC2	46.46 ± 1.05	45.60 ± 13.60	98.14%	2.31%

### Analysis of hair from an acute promyelocytic leukaemia patient treated with arsenic trioxide

The metabolism and detoxification of arsenic have been extensively studied by Vahter^17^. In the early phase following a single exposure, arsenic distributes to the liver and kidney. In the later phase, i.e., 24 h after the exposure event, little of the original amount remains in these organs, and the greatest amount of As can be found in the tissues that accumulate arsenic more readily, such as skin, hair and nails^18, 19^.

**Figure 4:**
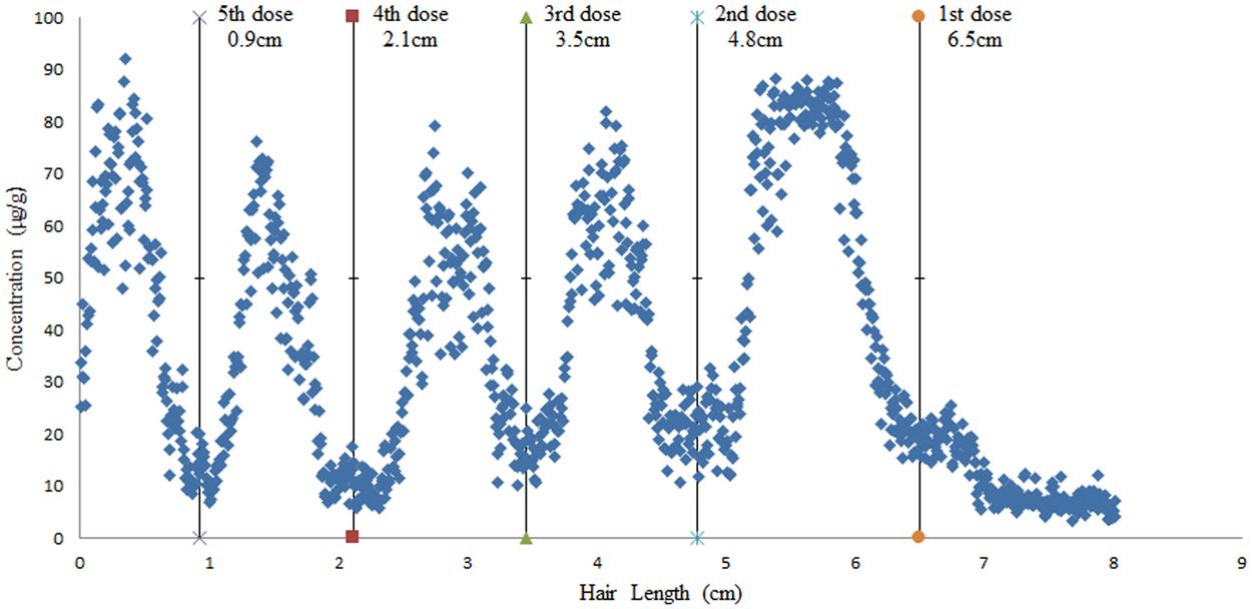
Concentration along a hair strand from a patient treated with medication five times, 10 mg As2O3 per day lasting 14 days for each course of treatment (except for the first administration).

The ability of this proposed method to provide spatial distribution information of As along the hair strands and infer the time at which the drug entered the body was tested. A hair sample collected from a volunteer who had been treated with arsenic trioxide (As_2_O_3_) in leukaemia and was not occupationally or environmentally exposed to As. This sample was entirely ablated from root to tip (total length of hair growth was 6.5 cm from the beginning of injecting As_2_O_3_).The hair sample was ablated by single spot scan mode. Dense spots can more clearly show the distribution of elements in the hair and more accurately infer the time of the drug entering the body. As shown in Fig. 4, informative As-distribution profiles of hair strands were obtained. LA-ICP-MS of the patient’s hair resolved five peaks corresponding to each dose of As_2_O_3_. The doses were 10 mg As_2_O_3_ and given once a day for 14 days (22 day for the first drug administration) in each course of treatment; the entire treatment process was 159 days. The average rate of hair growth was 0.04 cm per day, and the hair growth length during the period when drug was administered and hair was sampled, was 0.9 cm, 2.1 cm, 3.5 cm, 4.8 cm, and 6.5 cm, as shown in Fig. 4. According to the formula below, the estimated time since drug administration could be calculated. Good agreement was found between the estimated time and the actual time as shown in Table 3. This study demonstrates that our method using single spot scan mode may be used to monitor the exposure history over the most recent months and infer the time at which drug entered the body. Our study may help clinicians or forensic scientists to ascertain the degree of poisoning and determine the date of poisoning with relative accuracy.

$${\rm{Estimated}}\,\mathrm{time}({\rm{d}})=\frac{\mathrm{Length\; of\; hair\; growth}\,(\mathrm{cm})}{0.04\,(cm/d)}$$

**Table 3:** Comparison of actual and estimated time of drug entering body.

Time for the medicine	The number of days for using drug (day)	Length of hair growth^a^ (cm)	Actual time^b^ (day)	Estimated time^c^ (day)	Time of deviation (day)
September 30 2015	22	6.5	159	163	+4
November 7 2015	14	4.8	121	120	−1
December 7 2015	14	3.5	91	88	−3
January 4 2016	14	2.1	63	53	−10
February 4 2016	14	0.9	32	23	−9

^a^Length of hair growth during a period of using drug and sampling.

^b^The time interval between using drug and sampling.

^c^The time calculated by the formula.

## Conclusions

We created an analytical strategy of using LA-ICP-MS with matrix-matched calibration to quantify the concentration of arsenic and lead in hair. This quantification strategy would be most useful if no suitable reference materials were available, prohibiting the use of other techniques. Although this was not investigated in the present work, the approach could likely be used to quantify other trace elements along the hair strands. Our study showed that the arsenic distribution found in the analysed hair may contribute to the optimization of arsenic trioxide therapy. The proposed method can be employed in routine analysis, which can extend the use of hair analysis for therapy, occupational, nutritional and toxicological controls as well as in studying the time when drugs enter the body.
